# Supplementary material for: Variable opportunities for outcrossing result in hotspots of novel genetic variation in a pathogen metapopulation
Source: eLife. 2019 Jun 18;8:e47091. doi: 10.7554/eLife.47091 (PMC6667214; doi:10.7554/eLife.47091)
Supplement: Supplementary file 1. — MLG: MultiLocus Genotype. [file elife-47091-supp1.docx]

Supplementary FILE 1

**Table S1. Characteristics of the pathogen metapopulation for each year.** MLG: MultiLocus Genotype.

| **Year** | **Number of infected populations detected & sampled** | **Number of samples** | **Number of MLG** | **Number of new MLG (compared to the previous year)** | **Number of co-infected samples (%)** | **Number of populations with at least one co-infection (%)** |
| --- | --- | --- | --- | --- | --- | --- |
| 2012 | 619 | 4455 | 508 | NA | 893 (20%) | 346 (58%) |
| 2013 | 703 | 2945 | 501 | 182 | 769 (26%) | 366 (52%) |
| 2014 | 693 | 3591 | 517 | 189 | 766 (21%) | 344 (49%) |
| 2015 | 833 | 4474 | 571 | 235 | 861 (18%) | 394 (45%) |
